# Arterial tortuosity syndrome presenting as severe precapillary pulmonary hypertension in adulthood

**DOI:** 10.1016/j.ijcchd.2026.100683

**Published:** 2026-05-14

**Authors:** César Roberto Baltodano Dangla, Christopher Kaleb Romero Ríos, César Jeovany Urbina Maliaños, Mario Alberto Canales Reyes, Byron Larios Alemán, Ada Lila Lacayo Molina

**Affiliations:** a Department of Cardiology, Hospital Militar Escuela “Dr. Alejandro Dávila Bolaños”, Managua, Nicaragua; b School of Medicine, Universidad de Defensa de Nicaragua "4 de mayo". Managua, Nicaragua; c Department of Imagenology, Hospital Militar Escuela “Dr. Alejandro Dávila Bolaños”, Managua, Nicaragua

**Keywords:** Pulmonary hypertension, Pulmonary artery abnormalities, Connective tissue disorder, Right heart catheterization, Vascular tortuosity

## Abstract

**Background:**

Arterial tortuosity syndrome (ATS) is an ultra-rare hereditary connective tissue disorder characterized by elongation and extreme tortuosity of medium- and large-caliber arteries. Adult presentation is exceptional, and pulmonary arterial involvement as a cause of severe precapillary pulmonary hypertension is rarely reported.

**Case summary:**

A 47-year-old woman presented with progressive exertional dyspnea (WHO functional class II–III). Echocardiography and right heart catheterization confirmed severe precapillary pulmonary hypertension with markedly elevated pulmonary vascular resistance. Advanced vascular imaging and pulmonary angiography demonstrated bilateral pulmonary artery looping with dynamic functional stenoses, without fixed obstruction. Diffuse systemic arterial tortuosity and subsequent genetic testing, which identified a pathogenic variant in the *SLC2A10* gene, definitively confirmed the diagnosis of ATS**.**

**Conclusions:**

Dynamic pulmonary artery tortuosity can produce severe precapillary pulmonary hypertension despite preserved distal perfusion. ATS should be considered in unexplained cases with atypical pulmonary arterial anatomy.

## Highlights

• ATS in adults can present as severe precapillary pulmonary hypertension.
• Dynamic pulmonary artery looping causes severe functional obstruction.
• Advanced imaging and genetic testing are key for definitive ATS diagnosis.
• Dynamic narrowing alters management, deferring focal interventions.

## 1 Introduction

Arterial tortuosity syndrome (ATS) is an ultra-rare autosomal recessive connective tissue disorder characterized by elongation and pronounced tortuosity of medium- and large-caliber arteries, with an estimated prevalence below 1 per 1,000,000 live births [1,2]. Fewer than 120 genetically confirmed cases have been reported worldwide, the majority diagnosed in childhood, reflecting both the rarity of the condition and its early vascular expression [[1], [2], [3], [4]]. Adult presentation remains exceptional and is often driven by progressive cardiovascular complications rather than overt systemic features. This report describes an adult presentation of ATS manifesting as severe precapillary pulmonary hypertension caused by dynamic pulmonary artery looping, documented with invasive hemodynamics and multimodal imaging.

## 2 Case presentation

A 47-year-old woman was referred for evaluation of progressive exertional dyspnea over a 2-year period. Symptoms had gradually advanced to World Health Organization (WHO) functional class II–III limitation, restricting her ability to walk short distances. She denied syncope, chest pain, palpitations, hemoptysis, or peripheral edema. Physical examination revealed signs suggestive of right ventricular pressure overload without evidence of left-sided heart failure or systemic congestion. The patient reported a history of bronchial asthma during childhood and adolescence, but had no prior diagnosis of cardiovascular disease, thromboembolic events, connective tissue disorders, or relevant family history of vascular abnormalities.

**Fig. 1:**
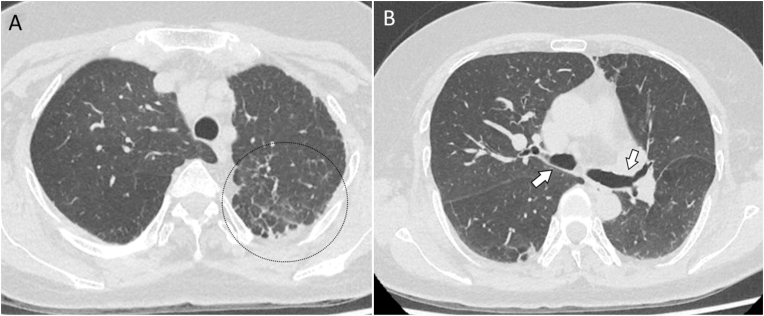
**High-Resolution CT Demonstrating Limited Parenchymal Disease and Bronchial Displacement** High-resolution computed tomography (lung window). (A) Focal interstitial abnormalities in the apical segment of the left upper lobe (dotted circle), insufficient to explain the severity of pulmonary hypertension. (B) Posterior displacement of the left main bronchus (right arrow) secondary to extrinsic compression from the tortuous left pulmonary artery (LPA). The right main bronchus (left arrow) follows a preserved anatomical course. These findings support a predominantly vascular rather than parenchymal mechanism.

**Fig. 2:**
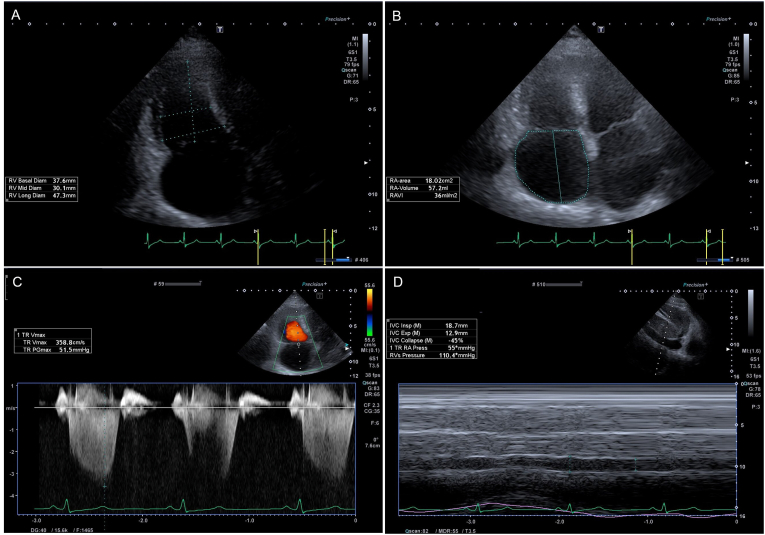
**Echocardiographic Evaluation of Right Heart Chambers and Pulmonary Hemodynamics** Transthoracic echocardiographic assessment. (A) Apical four-chamber view focused on the right ventricle (RV) demonstrating basal (37.6 mm) and mid-cavity (30.1 mm) diameters within normal limits according to ASE guidelines. (B) Quantification of the right atrium (RA) showing a right atrial volume index (RAVI) of 36 mL/m^2^, consistent with moderate chamber dilation. (C) Continuous-wave Doppler recording of the tricuspid regurgitation jet demonstrating a peak velocity of 3.58 m/s and a peak pressure gradient of 51.5 mmHg. (D) M-mode assessment of the inferior vena cava (IVC) showing a diameter of 18.7 mm with 45% inspiratory collapse, resulting in an estimated right ventricular systolic pressure (RVSP) of 110.4 mmHg.

Pulmonary function testing demonstrated mild obstructive changes without significant restriction or impaired diffusion capacity. High-resolution chest computed tomography revealed limited interstitial abnormalities that were insufficient to explain the severity of pulmonary hypertension (Fig. 1A). Notably, posterior displacement of the left main bronchus suggested extrinsic vascular compression (Fig. 1B), raising suspicion of an underlying vascular abnormality. Transthoracic echocardiography demonstrated moderate right atrial dilation with normal right ventricular dimensions, significant tricuspid regurgitation, and an estimated pulmonary artery systolic pressure of 110.4 mmHg, consistent with severe pulmonary hypertension (Fig. 2); left ventricular systolic function was preserved.

**Fig. 3:**
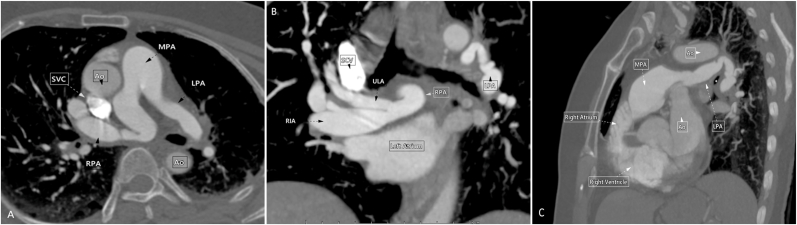
**CT Angiography Revealing Pulmonary Artery Tortuosity and Segmental Loop Formation** Multiplanar CT angiographic reconstruction. (A) Enlarged main pulmonary artery (MPA) bifurcating into tortuous right pulmonary artery (RPA) and left pulmonary artery (LPA). (B) The RPA exhibits marked looping prior to bifurcation into the upper lobar artery (ULA) and right interlobar artery (RIA), with caliber variation suggesting functional narrowing. (C) The LPA demonstrates looping over the left superior lobar bronchus (∗), causing mild extrinsic compression. Ao: Aorta; SVC: Superior Cava vein.

**Fig. 4:**
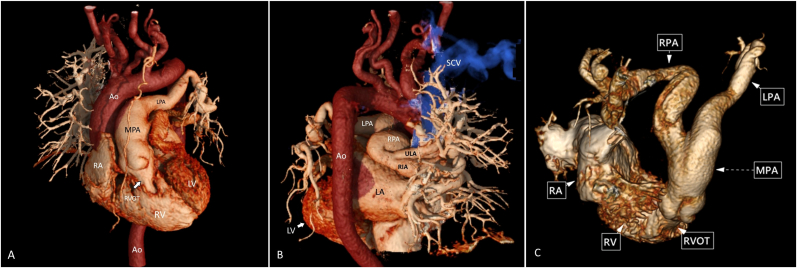
**Three-Dimensional Reconstruction Illustrating Pulmonary Artery Elongation and Looping** Three-dimensional volume-rendered reconstructions of CT angiography. (A) Coronal view demonstrating enlargement of the main pulmonary artery (MPA) relative to the right ventricular outflow tract (RVOT). (B) Posterior view highlighting right pulmonary artery **(**RPA) looping before its division into upper lobar artery (ULA) and right interlobar artery (RIA). (C) Oblique anterosuperior view with cardiac structures partially removed, emphasizing severe Right pulmonary artery (RPA) looping and marked left pulmonary artery (LPA) tortuosity. RA: Right atrium; RV: Right ventricle; LV: Left ventricle; Ao: Aorta; SCV: Superior Cava vein.

Given the discordance between imaging findings and clinical severity, right heart catheterization was performed. Hemodynamic assessment confirmed precapillary pulmonary hypertension, with a mean pulmonary artery pressure of 31 mmHg, normal pulmonary capillary wedge pressure, and markedly elevated pulmonary vascular resistance (12.5 Wood units). Subsequent computed tomography angiography revealed marked tortuosity of the main pulmonary artery and its branches, with loop formation and segmental caliber variation suggestive of functional narrowing (Fig. 3). Three-dimensional volume-rendered reconstructions further delineated pronounced elongation and looping of both pulmonary arteries (Fig. 4).

**Fig. 5:**
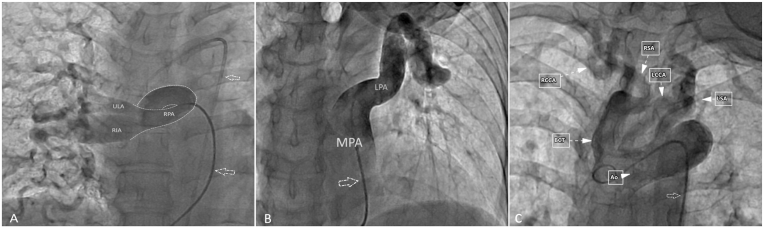
**Invasive Angiographic Confirmation of Dynamic Pulmonary Artery Looping and Functional Stenosis** Diagnostic pulmonary angiography. (A) The right pulmonary artery (RPA) (outlined) demonstrates a medial loop producing segmental narrowing while maintaining perfusion of the three right pulmonary lobes through the upper lobar artery (ULA) and right interlobar artery (RIA). of the right upper and interlobar branches. (B) The left pulmonary artery **(**LPA) shows a distal loop with reduced caliber and predominant perfusion of the left lower lobe. (C) Concomitant tortuosity of the supra-aortic vessels is visualized. The intravascular catheter is indicated by the dotted arrow.

**Fig. 6:**
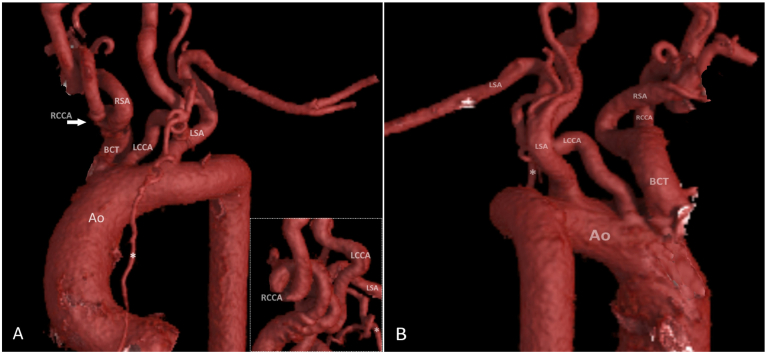
**Supra-Aortic Trunk Tortuosity Demonstrating Systemic Vascular Involvement** Three-dimensional CT angiographic reconstruction of the aortic arch and supra-aortic branches. (A) Left oblique-coronal view showing marked tortuosity of the right common carotid artery (RCCA) (dotted square). (B) Posterior oblique view demonstrating looping of both carotid and subclavian arteries from their origin at the aortic arch. These findings confirm diffuse systemic arterial involvement consistent with arterial tortuosity syndrome. Ao: Aorta; BCT: Brachiocephalic trunk; SVC: Superior cava vein; RCCA: Right common carotid artery; LCCA: Left common carotid artery; RSA: Right subclavian artery; RSV: Right subclavian vein ; ∗: Internal thoracic artery; LPA: Left pulmonary artery.

Diagnostic pulmonary angiography confirmed dynamic narrowing at these looping segments, generating significant translesional pressure gradients while maintaining distal perfusion (Fig. 5). Additional imaging demonstrated diffuse systemic arterial tortuosity involving the supra-aortic vessels (Fig. 6), as well as renal and iliac arteries. The diagnosis of Arterial Tortuosity Syndrome was definitively confirmed by genetic testing, which identified a pathogenic homozygous mutation in the SLC2A10 gene.

Given the predominantly dynamic nature of the pulmonary arterial obstruction and the absence of fixed critical stenosis amenable to focal intervention, surgical or percutaneous treatment was deferred. After multidisciplinary discussion, targeted pulmonary hypertension therapy with sildenafil was initiated. Supportive management included antiplatelet therapy and inhaled bronchodilators. The patient remained hemodynamically stable without procedural complications and reported symptomatic improvement at rest. Close outpatient follow-up was established to monitor right ventricular function, pulmonary hemodynamics, and overall clinical trajectory.

## 3 Discussion

Although ATS predominantly involves the systemic arterial tree, pulmonary arterial involvement has emerged as a clinically relevant yet underrecognized manifestation. Excessive elongation of the pulmonary arteries may lead to vessel kinking, looping, and segmental caliber variation, producing dynamic functional stenoses capable of increasing right ventricular afterload and ultimately causing precapillary pulmonary hypertension [1,5]. This mechanism differs fundamentally from fixed obstructive lesions observed in congenital pulmonary artery stenosis or chronic thromboembolic pulmonary hypertension, where structural luminal obstruction predominates.

In the present case, the severity of pulmonary hypertension was clearly disproportionate to the limited parenchymal lung abnormalities, prompting invasive hemodynamic evaluation. Right heart catheterization confirmed severe precapillary pulmonary hypertension with markedly elevated pulmonary vascular resistance, while pulmonary angiography demonstrated bilateral pulmonary artery looping associated with significant translesional pressure gradients. These findings strongly support dynamic functional obstruction as the principal pathophysiological mechanism. Importantly, such dynamic narrowing may be underestimated or incompletely characterized by noninvasive imaging modalities alone [5,6].

Current international guidelines for pulmonary hypertension emphasize pulmonary vascular remodeling, left heart disease, lung disease, and chronic thromboembolic mechanisms [7]. Structural arterial abnormalities such as those observed in genetically confirmed ATS are not specifically addressed, and therefore patients with ATS-related pulmonary hypertension may fall outside conventional diagnostic and therapeutic algorithms. This underscores the importance of integrating invasive hemodynamics with advanced vascular imaging when evaluating unexplained precapillary pulmonary hypertension.

From a therapeutic standpoint, no disease-modifying treatments exist for ATS. Management is therefore directed toward mitigating hemodynamic consequences and preventing vascular complications. Surgical, percutaneous, and hybrid approaches have been described in selected patients with focal or fixed pulmonary artery stenoses [6,8,9]. However, when obstruction is predominantly dynamic as occurs in arterial looping the benefit of intervention remains uncertain and must be carefully balanced against procedural risk. In such scenarios, targeted pulmonary hypertension therapy combined with close longitudinal surveillance may represent a prudent initial strategy.

This case broadens the adult phenotypic spectrum of ATS and highlights pulmonary artery tortuosity as a rare structural cause of precapillary pulmonary hypertension. Recognition of this mechanism is essential to avoid misclassification, refine diagnostic evaluation, and guide individualized management in a condition that remains insufficiently represented in contemporary guidelines.

## 4 Conclusions

Arterial tortuosity syndrome may present in adulthood as severe precapillary pulmonary hypertension caused by dynamic pulmonary artery looping. Identification of diffuse arterial tortuosity affecting both pulmonary and systemic circulations is a key diagnostic clue, with genetic testing providing definitive confirmation. Comprehensive evaluation integrating right heart catheterization and advanced vascular imaging is essential to define the underlying mechanism and guide individualized management in this rare condition.

## CRediT authorship contribution statement

**César Roberto Baltodano Dangla:** Conceptualization, Investigation, Supervision, Validation, Writing – original draft, Data curation, Formal analysis, Methodology, Project administration, Resources, Software, Visualization, Writing – review & editing. **Christopher Kaleb Romero Ríos:** Conceptualization, Data curation, Formal analysis, Investigation, Methodology, Project administration, Resources, Software, Supervision, Validation, Visualization, Writing – original draft, Writing – review & editing. **César Jeovany Urbina Maliaños:** Conceptualization, Investigation, Supervision, Validation, Writing – original draft. **Mario Alberto Canales Reyes:** Conceptualization, Supervision, Validation, Writing – original draft. **Byron Larios Alemán:** Conceptualization, Data curation, Investigation, Methodology, Validation, Visualization. **Ada Lila Lacayo Molina:** Conceptualization, Supervision, Validation, Visualization, Data curation, Methodology.

## Funding

This research did not receive any specific grant from funding agencies in the public, commercial, or not-for-profit sectors.

## Declaration of competing interest

The authors declare that they have no known competing financial interests or personal relationships that could have appeared to influence the work reported in this paper.
